# Association between kidney function, frailty and receipt of invasive management after acute coronary syndrome

**DOI:** 10.1136/openhrt-2024-002875

**Published:** 2024-10-09

**Authors:** Jemima Kate Scott, Thomas Johnson, Fergus John Caskey, Pippa Bailey, Lucy Ellen Selman, Abdulrahim Mulla, Ben Glampson, Jim Davies, Dimitri Papdimitriou, Kerrie Woods, Kevin O'Gallagher, Bryan Williams, Folkert W Asselbergs, Erik K Mayer, Richard Lee, Christopher Herbert, Stuart W Grant, Nick Curzen, Iain Squire, Rajesh Kharbanda, Ajay Shah, Divaka Perera, Riyaz S Patel, Keith Channon, Jamil Mayet, Amit Kaura, Yoav Ben-Shlomo

**Affiliations:** 1 Population Health Sciences, University of Bristol, Bristol, UK; 2 Richard Bright Renal Service, North Bristol NHS Trust, Westbury on Trym, UK; 3 NIHR Bristol Biomedical Research Centre, Bristol, UK; 4 Translational Health Sciences, University of Bristol, Bristol, UK; 5 Richard Bright Renal Service, North Bristol NHS Trust, Bristol, UK; 6 NIHR Imperial Biomedical Research Centre, London, UK; 7 NIHR Oxford Biomedical Research Centre, Oxford, UK; 8 NIHR Biomedical Research Centre at Guy's and St Thomas' NHS Foundation Trust and King's College, London, UK; 9 NIHR University College London Hospitals Biomedical Research Centre, London, UK; 10 UCL Institute of Health Informatics, London, UK; 11 University College London Institute of Health Informatics, London, UK; 12 Royal Marsden Hospital NHS Trust, London, UK; 13 Leeds Biomedical Research Centre, Leeds, UK; 14 Department of Cardiothoracic Surgery, NIHR Manchester Biomedical Research Centre, Manchester, UK; 15 NIHR Southampton Clinical Research Facility, Southampton, UK; 16 Department of Cardiovascular Sciences, University of Leicester, Leicester, UK; 17 Glenfield Hospital, Leicester, UK; 18 Cardiology, NIHR Biomedical Research Centre at Guy's and St Thomas' NHS Foundation Trust and King's College, London, UK; 19 Epidemiology and Public Health, NIHR University College London Hospitals Biomedical Research Centre, London, UK; 20 Department of Cardiovascular Medicine, NIHR Oxford Biomedical Research Centre, Oxford, UK

**Keywords:** Coronary Angiography, Acute Coronary Syndrome, Percutaneous Coronary Intervention

## Abstract

**Background:**

Reduced estimated glomerular filtration rate (eGFR) is associated with lower use of invasive management and increased mortality after acute coronary syndrome (ACS). The reasons for this are unclear.

**Methods:**

A retrospective clinical cohort study was performed using data from the English National Institute for Health Research Health Informatics Collaborative (2010–2017). Multivariable logistic regression was used to investigate whether eGFR<90 mL/min/1.73 m^2^ was associated with conservative ACS management and test whether (a) differences in care could be related to frailty and (b) associations between eGFR and mortality could be related to variation in revascularisation rates.

**Results:**

Among 10 205 people with ACS, an eGFR of <60 mL/min/1.73m^2^ was found in 25%. Strong inverse linear associations were found between worsening eGFR category and receipt of invasive management, on a relative and absolute scale. People with an eGFR <30 mL compared with ≥90 mL/min/1.73 m^2^ were half as likely to receive coronary angiography (OR 0.50, 95% CI 0.40 to 0.64) after non-ST-elevation (NSTE)-ACS and one-third as likely after STEMI (OR 0.30, 95% CI 0.19 to 0.46), resulting in 15 and 17 per 100 fewer procedures, respectively. Following multivariable adjustment, the ORs for receipt of angiography following NSTE-ACS were 1.05 (95% CI 0.88 to 1.27), 0.98 (95% CI 0.77 to 1.26), 0.76 (95% CI 0.57 to 1.01) and 0.58 (95% CI 0.44 to 0.77) in eGFR categories 60–89, 45–59, 30–44 and <30, respectively. After STEMI, the respective ORs were 1.20 (95% CI 0.84 to 1.71), 0.77 (95% CI 0.47 to 1.24), 0.33 (95% CI 0.20 to 0.56) and 0.28 (95% CI 0.16 to 0.48) (p<0.001 for linear trends). ORs were unchanged following adjustment for frailty. A positive association between the worse eGFR category and 30-day mortality was found (test for trend p<0.001), which was unaffected by adjustment for frailty.

**Conclusions:**

In people with ACS, lower eGFR was associated with reduced receipt of invasive coronary management and increased mortality. Adjustment for frailty failed to change these observations. Further research is required to explain these disparities and determine whether treatment variation reflects optimal care for people with low eGFR.

**Trial registration number:**

NCT03507309.

**WHAT IS ALREADY KNOWN ON THIS TOPIC**

- One in three people with acute coronary syndrome has reduced kidney function.
- Differences in care for acute coronary syndrome exist between people with and without impaired kidney function.

**WHAT THIS STUDY ADDS**

- People with reduced kidney function continue to be less likely to receive invasive management for acute coronary syndrome, on both a relative and absolute scale.
- Treatment variation appears to be driven by kidney function per se, rather than associated comorbidities or frailty.

**HOW THIS STUDY MIGHT AFFECT RESEARCH, PRACTICE OR POLICY**

- Clinicians should be aware that reduced use of invasive management in people with reduced kidney function and high-risk acute coronary syndrome may not represent best practice and contradicts current clinical guidelines.

## Introduction

 Up to 40% of people who suffer an acute coronary syndrome (ACS) have a reduced estimated glomerular filtration rate (eGFR <60 mL/min/1.73 m^2^).^1^ These individuals differ from those with normal eGFR (≥60). They have an increased ratio of non-ST-elevation ACS (NSTE-ACS) to ST-elevation myocardial infarction (STEMI), are older, more often female and have a greater burden of comorbid conditions.^1^ The majority of these variables can be identified and measured in routine healthcare datasets and thus accounted for in observational research.

People with reduced kidney function are more likely to receive conservative management after ACS and have increased mortality.^1^,^3^ Previous observational research has described reduced rates of invasive management,^2^,^4^ prescriptions of secondary preventative medications^5^ and referral to cardiac rehabilitation programmes^6^ in people with low eGFR, versus those with normal kidney function. Following ACS, in-hospital mortality in people with severely reduced eGFR (<30 mL/min/1.73 m^2^) is 4–5 times that of people with normal kidney function.^4^ Variation persists after adjustment for confounders including age, sex and comorbidity.

It remains unclear why, after ACS, low eGFR is associated with (1) more conservative care and (2) greater mortality. Variation in care with eGFR has been demonstrated in both NSTE-ACS and STEMI.^2 3^ Regarding STEMIs, it is unlikely that the immediate eGFR influences treatment decision-making, as this is rarely reported before time-critical treatment for STEMI is activated, though a history of kidney disease may be known. It also remains unclear whether reduced use of invasive management in people with low eGFR causally contributes to increased mortality.^2^

Frailty is a theoretically plausible mediator of the relationship between eGFR and invasive management after ACS. It is associated with reduced use of invasive management after ACS in the general population^7^ and with low eGFR.^8^ Clinicians’ assessments of frailty are known to influence clinical decisions.^9^,^11^ The close association between eGFR and frailty could explain how kidney function appears to influence STEMI management decisions without kidney test results being known.

In this study, we aimed to investigate (1) the association between reported eGFR and the receipt of invasive management after ACS and (2) whether frailty, a factor rarely accounted for in studies using routine healthcare datasets, explains any observed association between reduced eGFR and rates of invasive management and mortality, following ACS.

## Methods

### Study design and participants

We conducted a retrospective cohort study using data from five English hospital trusts within the National Institute for Health Research Health Informatics Collaborative (NIHR HIC) (online supplemental file 1). Eligible patients were diagnosed with ACS between 2010 (2008 for University College Hospital) and 2017. Follow-up was from the initial troponin result until death or censoring on 1 April 2017. Classification of ACS was made based on International Statistical Classification of Diseases and Related Health Problems (ICD-10) discharge codes in diagnostic positions 1 or 2 (online supplemental table 2). NSTE-ACS included unstable angina (UA) and NSTE MI. Where ACS was recorded in both positions 1 and 2, we classified the ACS according to position 1. Only the first hospital admission with a discharge diagnosis of ACS was eligible. We excluded people aged under 18 years, missing kidney function within 48 hours of the initial troponin test, and those with a coronary intervention that preceded the first troponin (online supplemental figure 1).

### Variables

#### Exposure

We calculated eGFR using the Chronic Kidney Disease (CKD) Epidemiology Collaboration equation without ethnicity. Where creatinine was not available, we used the eGFR reported in the NIHR HIC dataset. We categorised eGFR into (1) ≥90 mL/min/1.73 m^2^, (2) 60–89, (3) 45–59, (4) 30–44 and (5) <30, following the KDIGO CKD classification.^12^ Identification of people receiving kidney replacement therapy was made according to ICD-10 discharge diagnoses (online supplemental table 2), with the code in the lowest diagnostic position taking preference. Transplant recipients were classified according to eGFR and dialysis users as eGFR<30 mL/min/1.73 m^2^.

#### Covariables

Covariables included sex, age (5-year age groups), ethnicity (white vs other), smoking status (previous, current, never), coded obesity or a related disorder, prior diagnosis of or treatment for cardiovascular or cerebrovascular disease (CVD), diabetes mellitus and chronic obstructive pulmonary disease (COPD). Comorbidities were defined from ICD-10 discharge diagnoses (online supplemental table 2). We determined frailty category using the multimorbidity Frailty Index (mFI) for people 65 years or older (online supplemental table 3).^13^ We also used (1) the Hospital Frailty Risk Score (HFRS)^14^ and (2) the comorbidity count.

#### Outcomes

Our primary outcome was the receipt of inpatient coronary angiography with or without subsequent revascularisation. Secondary outcomes were^15^ inpatient revascularisation (percutaneous coronary intervention (PCI) or coronary artery bypass graft) in those who had received angiography, 30-day mortality and death during follow-up. We defined invasive management (angiography with or without revascularisation) as occurring during the index ACS admission. Vital status was ascertained using the National Patient Demographic Service.

### Statistical analysis

#### Main analyses

Continuous data were skewed so presented as medians with IQRs. Categorical data were summarised as frequency and percentages. We used logistic regression to estimate the odds ratios (OR, 95% CI) of invasive management and 30-day death following ACS, by eGFR category and stratified by ACS type. The absolute risk difference (ARD) is presented per 100 people and was calculated from the logistic regression model adjusted for age and sex. We used Cox regression to estimate the hazards of all-cause long-term (>30 days) death by eGFR category and stratified by ACS type.

To avoid the risk of immortal time bias, we excluded people with early mortality, defined as death within the guideline-suggested optimal time frame for coronary angiography (72 and 24 hours for NSTE-ACS and STEMI respectively) from analyses of rates of invasive management.^16^ We investigated potential ‘a priori’ effect modification within each model (online supplemental table 4).

To assess the impact of frailty on the relationship between eGFR category and revascularisation and mortality, we adjusted each of the above models for the mFI as a potential mediator.

#### Sensitivity analyses

We prespecified several sensitivity analyses to address potential sources of bias in our methods:

1. Different methods of adjusting for early death.
2. Adjustment for confounding using a propensity score (PS) (online supplemental table 5).
3. Multiple imputation of missing ethnicity data (online supplemental table 6).
4. Estimation of frailty using the HFRS^17^ or comorbidity count.^18^
5. Adjustment for clustering at the hospital level.

Inclusion of:

1. Items included in the composite CVD covariable as distinct covariables.
2. People with a first troponin result within 24 hours after a coronary intervention.
3. People with a code for revascularisation but no code for angiography.

Exclusion of:

1. People with a code for UA.
2. People with a code for revascularisation but not for angiography.

Lastly, given the large difference in age found between eGFR categories, we performed a post hoc stratified analysis to assess the impact of age on the association between eGFR category and rates of revascularisation.

Details of the methodology for these analyses are detailed in online supplemental table 7.

### Missing covariable data

We used the date of PCI to impute missing angiography dates, as all patients missing angiography had received PCI (2250 individuals). People were assumed to have received angiography if they had a code for revascularisation but none for angiography (300–4.7% of those revascularised). Individuals with missing ethnicity data were excluded from multivariable models in the main analyses (but are included in one of the sensitivity analyses). Statistical analyses were undertaken using Stata (V.16.0).

### Patient and public involvement

Members of the UK Renal Registry Patient Council highlighted cardiovascular disease care as a research priority for people with kidney disease (2018). A six-person patient involvement group brought together to oversee this, and related work will advise on methods of disseminating results to the patient community.

## Results

### Study population and baseline characteristics

Derivation of the study population is shown in online supplemental figure 1. Among the final sample of 10 205 people (6451 NSTE-ACS and 3754 STEMI), 25% had an eGFR <60 mL.min/1.73m^2^. 225 people (171 NSTE-ACS, 54 STEMI) were excluded from analyses of invasive management due to early mortality. Early mortality was progressively more common in those in lower eGFR categories (table 1). Ethnic category was missing in 1853 (18.2%) people.

**Table 1:** Table of characteristics by eGFR category

	Missing	eGFR* ≥90	eGFR 60–89	eGFR 45–59	eGFR 30–45	eGFR<30
	n (row %)	N=3397	N=4237	N=1149	N=753	N=669
ACS type	0					
STEMI		1568 (46.2%)	1516 (35.8%)	302 (26.3%)	197 (26.2%)	171 (25.6%)
NSTEMI		1326 (39.0%)	2034 (48.0%)	678 (59.0%)	437 (58.0%)	422 (63.1%)
UA		503 (14.8%)	687 (16.2%)	169 (14.7%)	119 (15.8%)	76 (11.4%)
Creatinine (µmol/L)	295† (2.9)	70 (62–77)	84 (73–94)	111 (96–122)	140 (124–159)	245 (194–366)
Age (years)	0	58 (51–64)	73 (65–81)	80 (72–86)	82 (75–88)	80 (71–87)
Female sex	0	640 (18.8%)	1391 (32.8%)	465 (40.5%)	321 (42.6%)	259 (38.7%)
Ethnicity	1853 (18.2)					
White		1885 (55.5%)	2716 (64.1%)	788 (68.6%)	485 (64.4%)	392 (58.6%)
Black		113 (3.3%)	143 (3.4%)	49 (4.3%)	27 (3.6%)	40 (6.0%)
Asian		419 (12.3%)	393 (9.3%)	85 (7.4%)	63 (8.4%)	98 (14.6%)
Mixed		277 (8.2%)	249 (5.9%)	43 (3.7%)	48 (6.4%)	39 (5.8%)
Smoking history	0					
Never smoked		1500 (44.2%)	2437 (57.5%)	774 (67.4%)	560 (74.4%)	515 (77.0%)
Ex smoker		573 (16.9%)	998 (23.6%)	250 (21.8%)	132 (17.5%)	98 (14.6%)
Current smoker		1324 (39.0%)	802 (18.9%)	125 (10.9%)	61 (8.1%)	56 (8.4%)
Diabetes mellitus	0	646 (19.0%)	901 (21.3%)	319 (27.8%)	256 (34.0%)	308 (46.0%)
CVD	0	2712 (79.8%)	3484 (82.2%)	960 (83.6%)	644 (85.5%)	587 (87.7%)
Hypercholesterolaemia	0	1212 (35.7%)	1545 (36.5%)	369 (32.1%)	221 (29.3%)	188 (28.1%)
Family history of IHD	0	928 (27.3%)	723 (17.1%)	109 (9.5%)	43 (5.7%)	35 (5.2%)
Arrhythmia	0	183 (5.4%)	495 (11.7%)	228 (19.8%)	176 (23.4%)	118 (17.6%)
Aortic stenosis	0	26 (0.8%)	96 (2.3%)	50 (4.4%)	40 (5.3%)	31 (4.6%)
CHF	0	249 (7.3%)	524 (12.4%)	253 (22.0%)	207 (27.5%)	223 (33.3%)
VTE	0	10 (0.3%)	15 (0.4%)	12 (1.0%)	8 (1.1%)	7 (1.0%)
COPD	0	110 (3.2%)	247 (5.8%)	93 (8.1%)	69 (9.2%)	61 (9.1%)
CVE	0	14 (0.4%)	33 (0.8%)	24 (2.1%)	10 (1.3%)	15 (2.2%)
Mental health disorder	0	1318 (38.8%)	758 (17.9%)	150 (13.1%)	75 (10.0%)	56 (8.4%)
Liver disease	0	20 (0.6%)	19 (0.4%)	6 (0.5%)	13 (1.7%)	12 (1.8%)
Malignancy	0	98 (2.9%)	244 (5.8%)	84 (7.3%)	64 (8.5%)	66 (9.9%)
Obesity	0	436 (12.8%)	427 (10.1%)	93 (8.1%)	58 (7.7%)	47 (7.0%)
Anaemia on admission	0	14 (0.4%)	20 (0.5%)	17 (1.5%)	14 (1.9%)	38 (5.7%)
Frailty	0					
Fit		2383 (88.5%)	2383 (88.5%)	432 (44.8%)	248 (39.8%)	203 (35.7%)
Mild frailty		272 (10.1%)	272 (10.1%)	403 (41.8%)	264 (42.4%)	241 (42.4%)
Moderate frailty		38 (1.4%)	38 (1.4%)	110 (11.4%)	91 (14.6%)	109 (19.2%)
Severe frailty		<5 (<1%)	<5 (<1%)	20 (2.1%)	20 (3.2%)	16 (2.8%)
Early mortality	0	12 (0.4%)	57 (1.3%)	41 (3.6%)	50 (6.6%)	65 (9.7%)

All continuous variables are presented as median (IQR). Categorical variables are presented as number (N) (%).

Missingness is 0 for most covariates as these are derived from ICD-10 codes: where the code was absent, the individual was assumed to be negative for the condition.

* eGFR presented in mlsL/min/1.73 m2.

† All 295 individuals for whom a creatinine value was missing from the available dataset, had an eGFR result reported within the same time frame (within 48 hours of the initial troponin test result).

ACSacute coronary syndromeCHF, congestive heart failure; COPD, chronic obstructive pulmonary disease; CVDcardiovascular diseaseCVE, cerebrovascular event; eGFRestimated glomerular filtration rateICD-10International Statistical Classification of Diseases, Tenth RevisionIHDischaemic heart diseaseNSTEMInon-STEMISTEMIST-elevation myocardial infarctionVTE, venous thrombo-embolism

Demographic characteristics and comorbid conditions are shown in table 1, stratified by eGFR category. People with lower eGFRs were more likely to be older, female and have prior CVD, congestive heart failure, diabetes mellitus and frailty, than those with a higher eGFR. They were less likely to be smokers and have hypercholesterolaemia or a family history of ischaemic heart disease.

### Invasive management outcomes

We found a strong inverse linear association between the worsening eGFR category and the receipt of angiography with or without revascularisation, after adjustment for either age and sex or multiple confounding variables (p<0.01 for all) (tables2^3^). Among those who had received angiography, there remained an inverse association between the worse eGFR category and receipt of revascularisation after NSTE-ACS, but not after STEMI.

**Table 2:** Age-adjusted and sex-adjusted odds of invasive management and absolute risk difference by eGFR category and ACS type

Outcome	ACS type	eGFR category	N (% of total)*	OR			Absolute risk difference		
				OR	95% CI	P value	ARD	95% CI	P value
Angiography with or without revascularisation	NSTE-ACS	≥90 mL/min/1.73 m^2^	1274 (70.1)	1.00			0.00		
		60–89	1662 (62.1)	1.07	0.92, 1.25	0.37	1.50	−1.78, 4.78	0.37
		45–59	421 (51.6)	0.94	0.76, 1.16	0.55	−1.37	−5.85, 3.12	0.55
		30–44	226 (43.6)	0.76	0.60, 0.96	0.02	−6.12	−11.47, 0.78	0.03
		<30	184 (40.8)	0.50	0.40, 0.64	<0.01	−15.37	−20.81, 9.93	<0.01
		Linear trend				<0.01			<0.01
	STEMI	≥90 mL/min/1.73 m^2^	1426 (91.0)	1.00			0.00		
		60–89	1333 (88.6)	0.92	0.69, 1.21	0.54	−0.81	−3.40, 1.78	0.54
		45–59	239 (81.9)	0.62	0.42, 0.93	0.02	−5.12	−9.78, 0.46	0.03
		30–44	131 (71.2)	0.38	0.25, 0.57	<0.01	−12.68	−19.21, 6.14	<0.01
		<30	102 (66.7)	0.30	0.19, 0.46	<0.01	−16.97	−24.49, 9.44	<0.01
		Linear trend				<0.01			<0.01
Revascularisation in those with angiography	NSTE-ACS	≥90 mL/min/1.73 m2	1148 (90.1)	1.00			0.00		
		60–89	1473 (88.6)	0.83	0.62, 1.09	<0.01	−1.81	−4.41, 0.78	0.17
		45–59	353 (83.9)	0.56	0.39, 0.81	<0.01	−6.34	−10.70, 1.98	<0.01
		30–44	177 (78.3)	0.39	0.26, 0.60	<0.01	−11.77	−17.95, 5.59	<0.01
		<30	149 (81.0)	0.45	0.29, 0.70	<0.01	−9.45	−15.62, 3.28	<0.01
		Linear trend				<0.01			<0.01
	STEMI	≥90 mL/min/1.73 m2	1335 (93.6)	1.00			0.00		
		60–89	1227 (92.1)	0.74	0.53, 1.04	<0.01	−2.00	−4.24, 0.25	0.08
		45–59	226 (94.6)	1.12	0.59, 2.13	0.73	0.62	−2.83, 4.07	0.73
		30–44	118 (90.1)	0.59	0.30, 1.16	0.13	−3.86	−9.64, 1.91	0.19
		<30	99 (97.10	2.13	0.64, 7.10	0.22	3.18	−0.56, 6.91	0.10
		Linear trend				0.32			0.32

* Number of people receiving the investigation or intervention (proportion of those potentially eligible).

ACSacute coronary syndromeARD, adjusted risk differenceeGFRestimated glomerular filtration rateNSTEnon-ST-elevationSTEMIST-elevation myocardial infarction

**Table 3:** Multivariable-adjusted odds of invasive management with and without adjustment for frailty

Outcome	ACS type	eGFR category	N (% of total)*	Fully adjusted†			Fully adjusted† with frailty score		
				OR	95% CI	P value	OR	95% CI	P value
Angiography with or without revascularisation	NSTE-ACS	≥90 mL/min/1.73 m^2^	1022 (69.5)	1.00			1.00		
		60–89	1372 (60.8)	1.05	0.88 to 1.27	0.58	1.06	0.88 to 1.27	0.56
		45–59	352 (50.9)	0.98	0.77 to 1.26	0.87	0.98	0.76 to 1.25	0.85
		30–44	184 (42.0)	0.76	0.57 to 1.01	0.06	0.76	0.57 to 1.01	0.06
		<30	164 (41.4)	0.58	0.44 to 0.77	<0.01	0.58	0.43 to 0.77	<0.01
		Linear trend				<0.01			<0.01
	STEMI	≥90 mL/min/1.73 m^2^	1111 (91.6)	1.00			1.00		
		60–89	1083 (90.4)	1.20	0.84 to 1.71	0.31	1.20	0.84 to 1.71	0.31
		45–59	199 (81.6)	0.77	0.47 to 1.24	0.28	0.77	0.47 to 1.24	0.28
		30–44	100 (69.0)	0.33	0.20 to 0.56	<0.01	0.33	0.20 to 0.56	<0.01
		<30	79 (63.7)	0.28	0.16 to 0.48	<0.01	0.28	0.16 to 0.49	<0.01
		Linear trend				<0.01			<0.01
Revascularisation in those with angiography	NSTE-ACS	≥90 mL/min/1.73 m^2^	925 (90.5)	1.00			1.00		
		60–89	1220 (88.9)	0.83	0.61 to 1.15	0.27	0.83	0.60 to 1.14	0.25
		45–59	294 (83.5)	0.56	0.37 to 0.86	0.01	0.58	0.38 to 0.89	0.01
		30–44	145 (78.8)	0.46	0.28 to 0.74	<0.01	0.49	0.30 to 0.79	<0.01
		<30	132 (80.5)	0.56	0.34 to 0.92	0.02	0.62	0.38 to 1.03	0.06
		Linear trend				<0.01			<0.01
	STEMI	≥90 mL/min/1.73 m^2^	1041 (93.7)	1.00			1.00		
		60–89	1004 (92.7)	0.93	0.63 to 1.38	0.72	0.94	0.63 to 1.40	0.77
		45–59	187 (94.0)	1.18	0.59 to 2.36	0.64	1.33	0.66 to 2.70	0.42
		30–44	91 (91.0)	0.8	0.35 to 1.81	0.59	0.89	0.39 to 2.04	0.79
		<30	78 (98.7)	6.79	0.89 to 51.53	0.06	8.08	1.05 to 62.44	0.05
		Linear trend				0.08			0.05

* Number of people receiving the investigation or intervention (proportion of those potentially eligible).

† Adjusted for age group, sex, ethnicity, obesity, prior CVD, COPD, diabetes mellitus and smoking group, with or without addition of frailty score.

COPDchronic obstructive pulmonary diseaseCVDcardiovascular diseaseNSTE-ACSnon-ST-elevation acute coronary syndromeSTEMIST-elevation myocardial infarction

### Non-ST-elevation acute coronary syndrome

**Figure 1:**
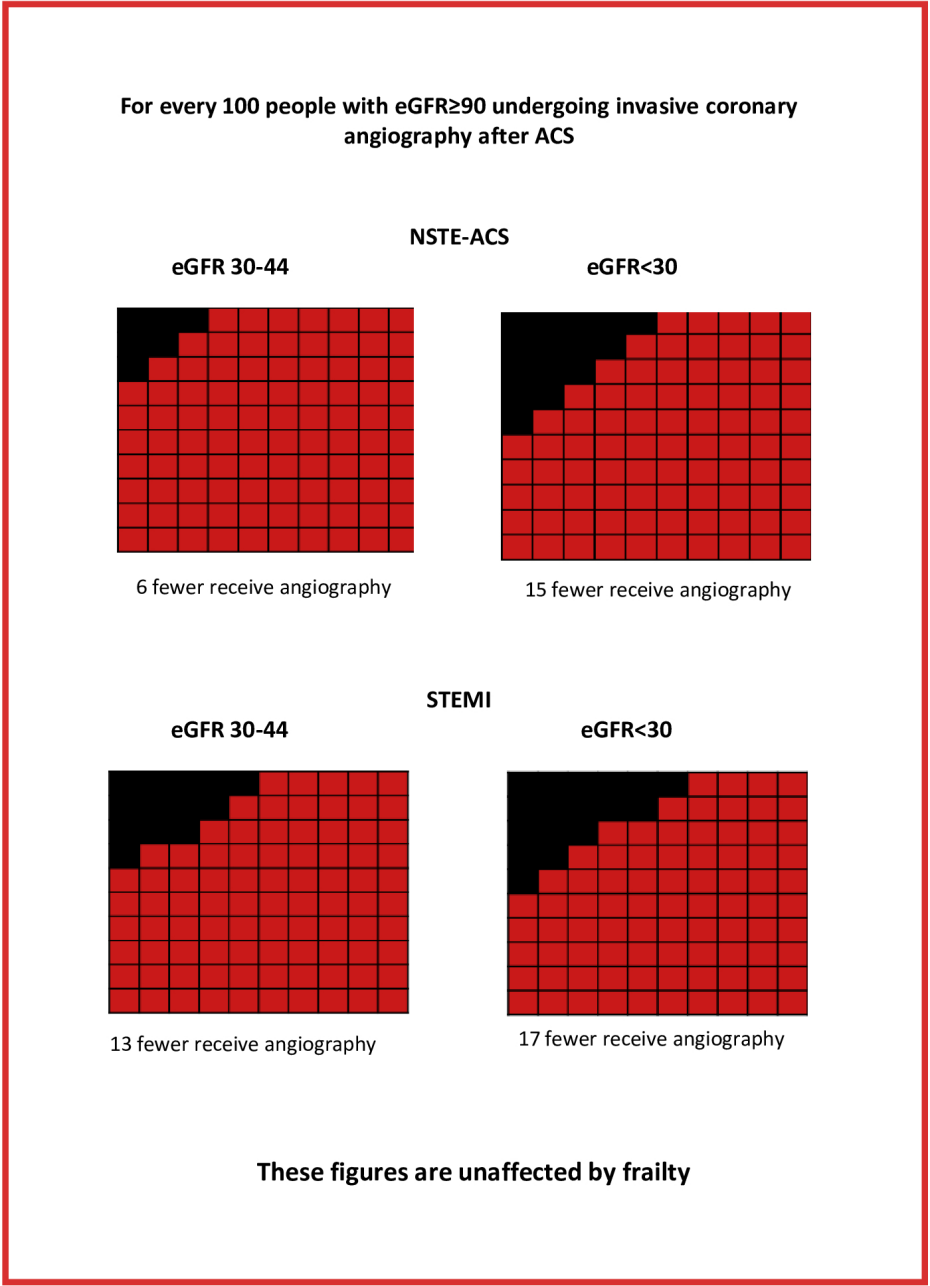
Absolute risk difference in receipt of coronary angiography after NSTE-ACS and STEMI, between people with and without reduced eGFR. eGFR, estimated glomerular filtration rate; NSTE-ACS, non-ST-elevation acute coronary syndrome; STEMI, ST-elevation myocardial infarction.

Following NSTE-ACS, people with an eGFR <30 mL/min/1.73 m^2^ were half as likely to undergo angiography as those with an eGFR ≥90 (OR 0.50 (95% CI 0.40, 0.64)) after adjustment for age and sex (table 2, figure 1).

After multivariable adjustment, individual effect estimates were slightly reduced (table 3). People with an eGFR<30 mL/min/1.73 m^2^ were less than two-thirds as likely to receive angiography as those with an eGFR ≥90. Among those who did receive angiography, people with an eGFR <60 or below were less likely to go to receive revascularisation, than those with an eGFR ≥90. Adjustment for frailty had little impact on effect estimates, refuting our hypothesis that reduced use of invasive management in people with kidney disease was due to the association between low eGFR and frailty.

### ST-elevation myocardial infarction

After adjustment for age and sex, people with an eGFR <30 were less than a third as likely to receive angiography than those with an eGFR ≥90 (table 2). Among those who received angiography, however, people with reduced eGFR were equally as likely to be revascularised as those with eGFR ≥90 (p=0.32). No meaningful difference in the effect estimates was seen after multivariable adjustment, with or without adjustment for frailty (table 3).

### Mortality outcomes

#### NSTE-ACS and STEMI

**Figure 2:**
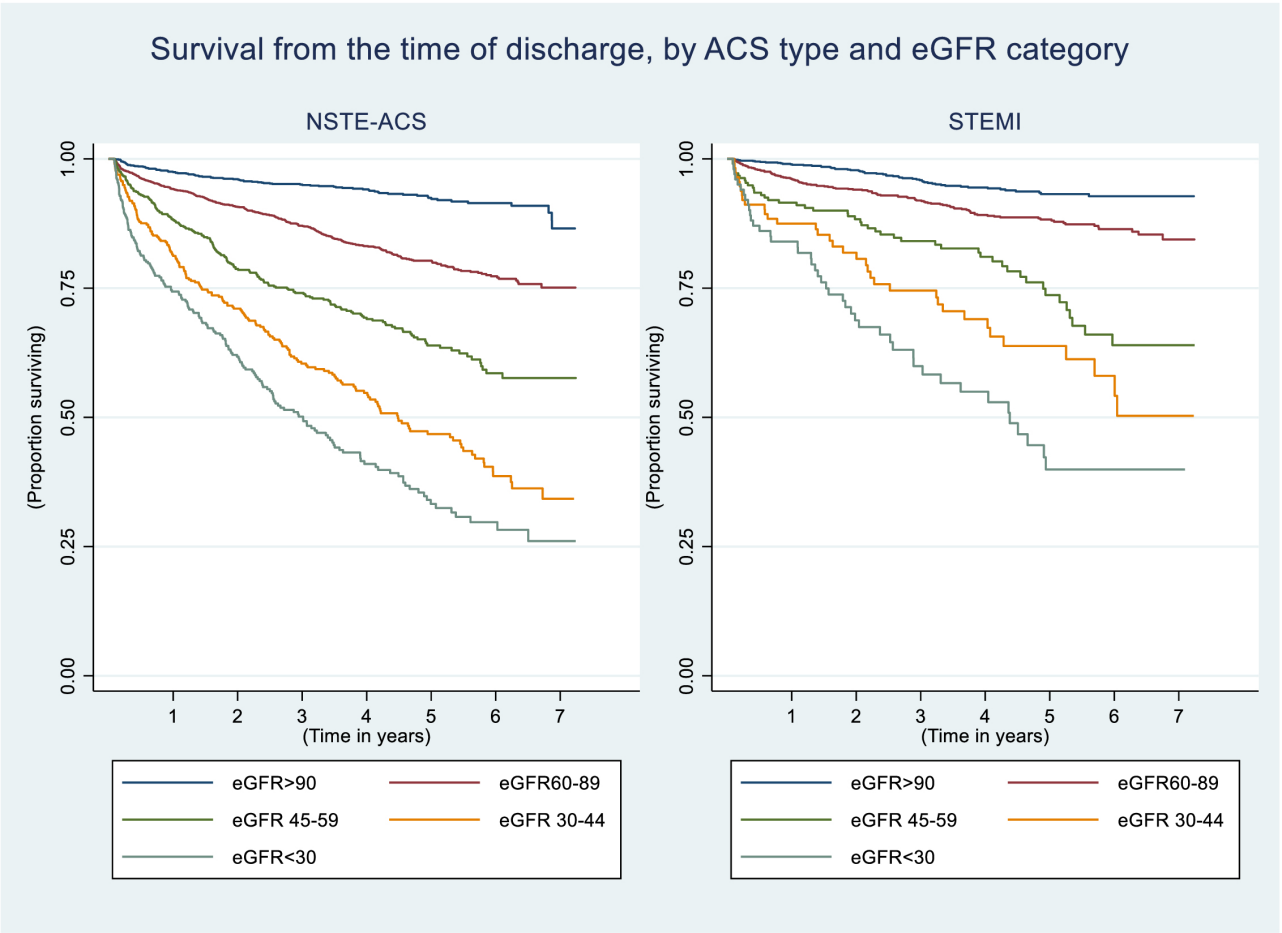
Kaplan-Meier curves demonstrating survival from the time of discharge after NSTE-ACS (left) and STEMI (right), by eGFR category. CKD, chronic kidney disease; eGFR, estimated glomerular filtration rate; NSTE-ACS, non-ST-elevation acute coronary syndrome; STEMI, ST-elevation myocardial infarction.

We found strong evidence of linear associations between eGFR category and mortality both up to, and beyond 30 days from ACS after adjustment for either age and sex or multiple confounding covariables (test for trend p<0.01 for all) (tables4  5). Compared with those with an eGFR ≥90 mL/min/1.73 m^2^, meaningful increases in 30-day mortality were seen in people with an eGFR below 90 mL/min/1.73 m^2^, and in long-term mortality in those with an eGFR <60 (tables4  5, figure 2), After multivariable adjustment, people with an eGFR <30 were 8-fold and 14-fold as likely to die within 30 days of NSTE-ACS or STEMI, respectively, than those with an eGFR ≥90 (table 5).

**Table 4:** Age-adjusted and sex-adjusted odds and absolute risk difference of short and long-term mortality by eGFR category and ACS type

Outcome	ACS type	eGFR category	N (% of total)*	Age and sex adjusted			Absolute risk difference		
				OR/HR	95% CI	P value	ARD	95% CI	P value
30-day death†	NSTE-ACS	≥90 mL/min/1.73 m^2^	34 (1.9)	1.00			0.00		
		60–89	125 (4.6)	1.63	1.06 to 2.51	0.03	1.62	0.34 to 2.90	0.01
		45–59	88 (10.4)	3.48	2.19 to 5.54	<0.01	6.02	3.85 to 8.19	<0.01
		30–44	87 (15.7)	5.44	3.38 to 8.74	<0.01	10.22	7.21 to 13.23	<0.01
		<30	97 (19.5)	7.62	4.81 to 12.1	<0.01	14.42	10.94 to 17.89	<0.01
		Linear trend				<0.01			<0.01
	STEMI	≥90 mL/min/1.73 m^2^	29 (1.9)	1.00			0.00		
		60–89	68 (4.5)	1.62	0.99 to 2.63	0.05	1.46	0.06 to 2.86	0.04
		45–59	44 (14.6)	5.36	3.10 to 9.27	<0.01	9.35	5.64 to 13.05	<0.01
		30–44	60 (30.5)	13.45	7.79 to 23.2	<0.01	22.27	15.95 to 28.59	<0.01
		<30	50 (29.2)	12.77	7.28 to 22.40	<0.01	21.3	14.75 to 27.94	<0.01
		Linear trend				<0.01			<0.01
Death after 30 days‡	NSTE-ACS	≥90 mL/min/1.73 m^2^	115 (6.4)	1.00			0.00		
		60–89	400 (15.4)	1.06	0.83 to 1.35	0.64	0.19	−0.62 to 1.01	0.64
		45–59	219 (28.9)	1.64	1.25 to 2.14	<0.01	2.08	0.36 to 3.80	0.02
		30–44	206 (43.9)	2.35	1.78 to 3.10	<0.01	4.41	1.30 to 7.51	<0.01
		<30	215 (53.6)	4.49	3.44 to 5.86	<0.01	11.37	3.95 to 18.78	<0.01
		Linear trend				<0.01			<0.01
	STEMI	≥90 mL/min/1.73 m^2^	60 (3.9)	1.00			0.00		
		60–89	144 (9.9)	1.20	0.84 to 1.72	0.32	0.55	−0.63 to 1.73	0.36
		45–59	57 (22.1)	2.09	1.35 to 3.23	<0.01	3.00	−0.28 to 6.28	0.07
		30–44	40 (29.2)	2.54	1.57 to 4.10	<0.01	4.24	−0.30 to 8.77	0.07
		<30	49 (40.5)	4.73	3.02 to 7.40	<0.01	10.26	0.47 to 20.05	0.04
		Linear trend				<0.01			<0.01

* Number of people receiving the investigation or intervention (proportion of those potentially eligible).

† Expressed as odds ratio (OR)OR.

‡ Expressed as hazard ratio (HR)HR.

ACSacute coronary syndromeARD, absolute risk differenceeGFRestimated glomerular filtration rateNSTEnon-ST-elevationSTEMInon-ST-elevation myocardial infarction

**Table 5:** Multivariable-adjusted odds of short and long-term mortality by eGFR category and ACS type, with and without adjustment for either frailty score or revascularisation status

Outcome	ACS type	eGFR category	N (% of total)*	Fully adjusted†			Fully adjusted† with frailty score		
				OR/HR	95% CI	P value	OR/HR	95% CI	P value
30-day death‡	NSTE-ACS	≥90 mL/min/1.73 m^2^	27 (1.8)	1.00			1.00		
		60–89	98 (4.3)	1.63	1.01 to 2.64	0.05	1.64	1.01 to 2.66	0.04
		45–59	68 (9.5)	3.45	2.04 to 5.82	<0.01	3.43	2.03 to 5.80	<0.01
		30–44	75 (15.9)	6.04	3.56 to 10.27	<0.01	6.00	3.53 to 10.21	<0.01
		<30	83 (19.2)	8.08	4.79 to 13.63	<0.01	7.91	4.68 to 13.37	<0.01
		Linear trend				<0.01			<0.01
	STEMI	≥90 mL/min/1.73m^2^	16 (1.3)	1.00			1.00		
		60–89	53 (4.4)	2.35	1.26 to 4.38	0.01	2.40	1.29 to 4.47	0.01
		45–59	34 (13.6)	7.09	3.57 to 14.08	<0.01	6.72	3.37 to 13.42	<0.01
		30–44	39 (25.7)	16.13	7.96 to 32.69	<0.01	15.23	7.47 to 31.04	<0.01
		<30	35 (25.7)	14.88	7.22 to 30.67	<0.01	13.94	6.71 to 28.99	<0.01
		Linear trend				<0.01			<0.01
Death after 30 days§	NSTE-ACS	≥90 mL/min/1.73m^2^	92 (6.3)	1.00			1.00		
		60–89	364 (16.6)	1.18	0.90 to 1.54	0.22	1.19	0.91 to 1.55	0.20
		45–59	200 (30.9)	1.77	1.32 to 2.37	<0.01	1.72	1.28 to 2.31	<0.01
		30–44	190 (48.0)	2.59	1.92 to 3.51	<0.01	2.51	1.85 to 3.40	<0.01
		<30	192 (55.1)	4.62	3.44 to 6.22	<0.01	4.41	3.27 to 5.94	<0.01
		Linear trend				<0.01			<0.01
	STEMI	≥90 mL/min/1.73m^2^	56 (4.7)	1.00			1.00		
		60–89	118 (10.2)	1.04	0.71 to 1.53	0.83	1.04	0.71 to 1.53	0.83
		45–59	48 (22.2)	1.79	1.13 to 2.86	0.01	1.74	1.09 to 2.77	0.02
		30–44	36 (31.9)	2.09	1.25 to 3.49	0.01	1.98	1.18 to 3.33	0.01
		<30	45 (44.6)	4.13	2.55 to 6.70	<0.01	4.03	2.48 to 6.56	<0.01
		Linear trend				<0.01			<0.01

* Number of people receiving the investigation or intervention (proportion of those potentially eligible).

† Adjusted for age group, gender, ethnicity, obesity, prior CVD, COPD, diabetes mellitus and smoking group.

‡ Expressed as odds ratio (OR)OR.

§ Expressed as hazard ratio (HR)HR.

COPDchronic obstructive pulmonary diseaseCVDcardiovascular diseaseeGFRestimated glomerular filtration rateNSTE-ACSnon-ST-elevation acute coronary syndromeSTEMIST-elevation myocardial infarction

We tested the hypothesis that increased mortality in those with reduced eGFR may be attributable to higher levels of frailty. However, we found little difference in effect estimates for either NTE-ACS or STEMI following additional adjustment for the mFI.

### Sensitivity analyses

People excluded due to lack of kidney function testing (67 people (0.7%)) were, on average, younger and had fewer comorbidities, than the study population (online supplemental table 8). Effect estimates for STEMI were slightly attenuated towards the null following adjustment for PS (online supplemental table 10). Exclusion of people with UA from the NSTEMI cohort (n=1625) resulted in a reduction in ORs for angiography in people with eGFR<60 mL/min/1.73 m^2^ (eg, eGFR <30 (OR 0.58 (95% CI 0.44 to 0.77) to 0.40 (95% CI 0.29 to 0.56)) (online supplemental table 17). Other prespecified sensitivity analyses had minimal impact on effect estimates and are presented in online supplemental tables 9 and 11–16.

We reanalysed receipt of revascularisation stratified by age group (<65, 65–75 or >75 years). For NSTEMI, the inverse associations were seen consistently across all age groups but for STEMI, the data suggested a potential qualitative interaction (p value for effect modification <0.001) between the age group and eGFR category (online supplemental table 18). However, the baseline group had very few observations, which may have artefactually affected the effect estimates. The analysis was, therefore, repeated after combining ‘eGFR 60–90’ with the ‘eGFR ≥90 mL’ group and the expected inverse association between worsening kidney function and reduced receipt of revascularisation was again evident (online supplemental table 19).

## Discussion

We observed strong inverse linear associations between worsening eGFR category and the receipt of invasive management following both NSTE-ACS and STEMI, on a relative and absolute scale. For example, following NSTE-ACS, people with an eGFR <30 mL compared with ≥90 mL/min/1.73 m^2^ were half as likely to receive angiography (OR 0.50, 95% CI 0.40, 0.64), resulting in 15 per 100 fewer procedures. Clinically significant reductions in rates of invasive management were not limited to those with the worst kidney function but there was in general a dose-response pattern across the range of function from eGFR <60 mL/min/1.73 m^2^. The disparities we identified did not appear to be driven by the increased prevalence of frailty among those with reduced kidney function.

Reduced kidney function has been associated with less use of invasive management in previous research.^1 4^ Our work adds to and extends these observations by testing for the possible mediating role of frailty status. This is, to our knowledge, a variable that has not been considered previously and could theoretically mediate the association between eGFR and invasive management. The inability of frailty to explain the observed associations suggests that kidney function per se, rather than associated demographics or health state, affects receipt of invasive management after ACS. Potential explanations include the fear of causing contrast nephropathy,^19^ vascular access difficulties and uncertainty regarding the mortality benefits of invasive management in people with low eGFR, whose risks may be compounded by coexistent anaemia or reduced left ventricular function.^20^ ^21^ ^5^ Attempts to lower the risk of contrast nephropathy by administering intravenous fluids or awaiting resolution of AKI may explain the reduced timeliness of angiography in people with low eGFR after NSTE-ACS. These findings are harder to explain in STEMI, where the urgency of intervention would be expected to supersede concerns about eGFR.

When we restricted our analysis to individuals who had received angiography, reduced eGFR continued to be associated with lower odds of revascularisation following NSTE-ACS, but not STEMI. For people with STEMI, the key decision regarding invasive management appears to be angiography, as this is almost always followed by immediate PCI. For those with NSTE-ACS, further decision-making occurs regarding revascularisation. Compared with those with normal kidney function, people with NSTE-ACS and reduced eGFR may be more likely to develop troponin elevation either without angiographically detected disease or with extensive disease not amenable to revascularisation.

We also observed strong linear associations between eGFR category and mortality after ACS; an increased risk of death before and after 30 days from ACS was seen in people with an eGFR <90 mL/min/1.73 m^2^. Again, variation in rates of frailty appeared to explain little of these associations. Inverse associations between kidney function and mortality have been described previously.^1 2 4^ People with low eGFR (especially with proteinuria) have higher baseline rates of all-cause and cardiovascular mortality, than those with normal eGFR.^22^ Those with ACS experience accelerated progression to end-stage kidney failure,^23^ and a greater risk of heart failure and recurrent ACS.^24^ ^25^

### Strengths and limitations

Our study had many strengths, including a sample size of over 10 000 people, and the identification of kidney impairment via biochemistry, which is preferable to the use of diagnostic codes.^14^ We used cost-effective real-world, routinely collected data and performed numerous sensitivity analyses. However, we recognise limitations associated with our data. First, the use of routine healthcare records prevented us from (1) assessing for potential confounding and mediation by socioeconomic position, left ventricular ejection fraction, severity of comorbidities, pharmacotherapy and/or individual healthcare preferences^26^ ; (2) being able to differentiate between acute and chronic kidney disease (CKD); (3) identifying type two MI^27^ and (4) Using a performance-based frailty measure (eg, grip strength). Although the mFI has not been validated in UK data, we chose this score as it is applicable in younger people than others calculable from NIHR HIC codes: Frailty is common in people with CKD as young as 65 years.^8^ Neither objective measures nor calculated frailty scores may reflect physicians’ subjective frailty assessments.

Second, due to low numbers, we decided to (1) include people receiving dialysis with those with eGFR <30 mL/min/1.73 m^2^, and kidney transplant recipients with their respective eGFR categories^3^; (2) categorise people in ethnic groups other than white as a single group while acknowledging this is a heterogeneous population and (3) we were not powered to fully exclude interactions between frailty and eGFR category.

Third, our methodology may have introduced limitations, such as (1) potential exaggeration of the association between eGFR and angiography following STEMI, as repeating the analysis using a PS attenuated this association. The qualitative message was, however, unchanged. (2) We excluded people with missing ethnicity data from multivariable models, however, no meaningful change was demonstrated when ethnicity was estimated using multiple imputation. (3) We assumed the pattern of missingness was missing at random and multiple imputation may have been biased if this was incorrect.

Further research is needed to explain what drives treatment disparities between people with and without reduced eGFR, to determine if there is inequity in care. Analysis of a more granular quantitative dataset would show whether receipt of other aspects of ACS care (further investigations, pharmacotherapy, outpatient follow-up) is also associated with kidney function, as well as the relationship between angiography findings and revascularisation in people with and without reduced eGFR. Qualitative research with patients and clinicians would contribute to our understanding of why differences exist, via the investigation of (a) treatment decision-making, (b) how risks and benefits of invasive management are deliberated and (c) the involvement and wishes of patients regarding treatment decisions.

## Conclusions

In our analysis of multicentre routine healthcare data from England, we observed clinically meaningful reductions in the receipt of invasive management following ACS for people with low eGFR despite adjustment for differences in frailty in addition to comorbidity, demographics and early death. Differences in rates of frailty failed to explain the increased mortality following ACS experienced by people with reduced eGFR. Kidney function per se, therefore, appears to drive both differential receipt of invasive management for ACS and mortality. Understanding the reasons for treatment variation may help to conclude whether these differences represent equitable and optimal ACS care for the high-risk kidney disease population.

### Preregistered clinical trial number

The preregistered clinical trial number is NCT03507309.

## supplementary material

10.1136/openhrt-2024-002875 online supplemental file 1

## Data Availability

Data may be obtained from a third party and are not publicly available.
