# Supplementary material for: Association between kidney function, frailty and receipt of invasive management after acute coronary syndrome
Source: Open Heart. 2024 Oct 9;11(2):e002875. doi: 10.1136/openhrt-2024-002875 (PMC11474759; doi:10.1136/openhrt-2024-002875)

Supplementary materials

Contents

Table of Contents

Contents 1

Tables 2

Supplementary table 1: English Hospital Trusts hosting NIHR Biomedical Research Centres contributing data towards the National Institute for Health Research Health Informatics Collaborative 2008-2017........ 2

Supplementary table 2: Diagnostic terms relating to ICD-10 diagnostic codes. 3

Supplementary table 3. ICD-v10 items included in the Multimorbidity Frailty index (v10) and Hospital Frailty Risk Score. 4

Supplementary table 4: Covariables identified "a priori" as potential effect modifiers of the association between low eGFR and reduced use of invasive management after ACS due to their potential causative association with eGFR, coronary intervention and death........ 9

Supplementary table 5: Covariables included in the propensity score. 9

Supplementary table 6: Covariates included in the multiple imputation model. 9

Supplementary table 7: Detailed methodology for sensitivity analyses designed to investigate potential sources of bias in the association between eGFR category and coronary angiography following ACS: 10

Supplementary table 8: Table of characteristics for patients for whom a kidney test result was available (included in main analyses) and those excluded from analyses due to lack of available kidney test result. 12

Supplementary table 9: Multivariable-adjusted odds of inpatient angiography by eGFR category and ACS type using different methods of adjustment for the competing risk of death. 13

Supplementary table 10. Comparison of effect estimates for the odds of inpatient angiography for people with eGFR<60mls/min/1.73m^2 versus those with an eGFR≥60 between multivariable and propensity score adjusted models, by ACS type........ 14

Supplementary table 11: Comparison of the multivariable-adjusted odds of inpatient angiography by eGFR category and ACS type using a complete case analysis versus multiple imputation of missing ethnicity data. 15

Supplementary table 12: Multivariable-adjusted odds of inpatient angiography by eGFR category and ACS type, with adjustment for Multimorbidity frailty index, Hospital frailty risk score or comorbidity count........ 16

Supplementary table 13: Multivariable-adjusted odds of inpatient angiography by ACS type, with and without adjustment for clustering at the hospital level. 17

Supplementary table 14: Multivariable-adjusted odds ratios for inpatient angiography adjusted for composite CVD variable versus multiple distinct CVD-related variables 18

Supplementary table 15: Multivariable-adjusted odds ratios for inpatient angiography without and with inclusion of people with a first troponin value recorded within the 24 hours following a coronary intervention. 19
Supplementary table 16: Multivariable-adjusted odds of angiography by eGFR category and ACS type, with and without inclusion of patients with a code for revascularization but not for angiography 19
Supplementary table 17: Multivariable-adjusted odds ratios for inpatient angiography and revascularisation by eGFR category for people with NSTE-ACS with and without inclusion of those with unstable angina........ 20
Supplementary table 18: Multivariable-adjusted odds ratios for revascularisation by eGFR category and ACS type, stratified by age group........ 21
Supplementary table 19: Multivariable-adjusted odds ratios for revascularisation after STEMI comparing eGFR>60mls/min/1.73m2 to eGFR categories <60, stratified by age group. 21
Supplementary table 19: STROBE Reporting Checklist 22
Figures 25
Supplementary figure 1: Flow chart of inclusion/exclusion 25

Tables

Supplementary table 1: English Hospital Trusts hosting NIHR Biomedical Research Centres contributing data towards the National Institute for Health Research Health Informatics Collaborative 2008-2017.

The NIHR HIC is a partnership of NHS Trusts, Organisations and Health Boards, including those hosting NIHR Biomedical Research Centres, designed to facilitate the equitable re-use of National Health Service data for translational research. Routine clinical data is extracted from the electronic health systems of contributing NHS Trusts (Five Trusts at the time of this data extract). The NIHR HIC Cardiovascular dataset employed in this analysis contains clinical data on all patients who underwent troponin testing within 24 hours of hospital admission. We then selected individuals from this dataset who had had a diagnosis of ACS confirmed according to the discharge diagnosis (in positions one or two).

**Healthcare Trusts**
Imperial College Healthcare, London<br>University College Hospital, London<br>Oxford University Hospitals, Oxford<br>King’s College Hospital, London

Guys & St Thomas' Hospital, London

Supplementary table 2: Diagnostic terms relating to ICD-10 diagnostic codes.

Diagnosis	Diagnostic terms from ICD-10
Aortic stenosis	Aortic (valve) stenosis; Aortic (valve) stenosis with insufficiency
Arrythmia	Atrial fibrillation and flutter, Supraventricular tachycardia, Ventricular tachycardia, Ventricular fibrillation, Atrioventricular block
Cardiac failure	Congestive heart failure, Left ventricular failure, Heart failure unspecified, Hypertensive heart disease with (congestive) heart failure
Cardiovascular disease	Old myocardial infarction, Peripheral vascular disease unspecified, Other specified peripheral vascular diseases, Atherosclerosis of arteries of extremities, Thoracic aortic aneurysm without mention of rupture, Abdominal aortic aneurysm without mention of rupture, Cerebral infarction unspecified, Other cerebral infarction, Cerebral infarction due to unspecified occlusion or stenosis of cerebral arteries, Cerebral infarction due to embolism of cerebral arteries, Cerebral infarction due to thrombosis of cerebral arteries, Sequelae of cerebral infarction, Intracerebral haemorrhage in brain stem, Intracerebral haemorrhage unspecified, Intracerebral haemorrhage in cerebellum, Congestive heart failure, Left ventricular failure, Heart failure unspecified, Hypertensive heart disease with (congestive) heart failure, Angina pectoris unspecified, Other forms of angina pectoris, Primary (essential) hypertension, Essential (primary) hypertension, Hypertensive renal disease with renal failure, Hypertensive heart and renal disease, Hypertension secondary to other renal disease, Hypertensive renal disease with renal failure, Hypertensive heart disease with (congestive) heart failure, Atherosclerotic heart disease; Chronic ischaemic heart disease, Personal history of disorders of the circulatory system, Coronary angioplasty implant, Presence of aortocoronary bypass graft, Transient cerebral ischaemic attack unspecified, Amaurosis fugax
Chronic obstructive pulmonary disease	Emphysema unspecified, Unspecified chronic bronchitis, Chronic obstructive pulmonary disease unspecified, Chronic obstructive pulmonary disease with acute lower respiratory infection, Chronic obstructive pulmonary disease with acute exacerbation unspecified
Diabetes mellitus	Diabetic polyneuropathy, Non-insulin dependent diabetes mellitus, Diabetic retinopathy, Diabetic mononeuropathy, Insulin-dependent diabetes mellitus, Unspecific diabetes mellitus, Glomerular disorders in diabetes mellitus, Other specific diabetes mellitus
Family history of ischaemic heart disease	Family history of ischaemic heart disease
Haemorrhagic cerebrovascular event	Intracerebral haemorrhage in brain stem, Intracerebral haemorrhage unspecified, Intracerebral haemorrhage in cerebellum
Hypercholesterolaemia	Pure hypercholesterolaemia
Ischaemic cerebrovascular event	Cerebral infarction unspecified, Other cerebral infarction, Cerebral infarction due to unspecified occlusion or stenosis of cerebral arteries, Cerebral infarction due to embolism of cerebral arteries, Cerebral infarction due to thrombosis of cerebral arteries, Sequelae of cerebral infarction
Ischaemic heart disease	Atherosclerotic heart disease; Chronic ischaemic heart disease, Personal history of disorders of the circulatory system, Coronary angioplasty implant, Presence of aortocoronary bypass graft

Liver disease	Alcoholic liver disease unspecified, Liver disease unspecified, Autoimmune hepatitis, Chronic viral hepatitis C, Acute hepatitis B without delta-agent and without hepatic coma, Acute hepatitis C, Chronic hepatitis unspecified, , Fatty (change of) liver not elsewhere classified, Other and unspecified cirrhosis of liver, Cystic disease of liver, Alcoholic cirrhosis of liver, Chronic hepatic failure, Hepatic failure unspecified, Oesophageal varices with bleeding in diseases classified elsewhere, Oesophageal varices without bleeding,
Malignancy	Malignant neoplasm of X, Personal history of malignant neoplasm of X, Malignant neoplasm: X, Secondary and unspecified malignant neoplasm: X, Secondary malignancy neoplasm of X, Chronic lymphocytic leukaemia of B-cell type, Chronic myeloid leukaemia, Non-Hodgkin lymphoma unspecified, B-cell lymphoma unspecified, Hodgkin lymphoma unspecified, Other classical Hodgkin lymphoma, Diffuse large B-cell lymphoma
Mental health disorder	Depressive episode unspecified, Mixed anxiety and depressive disorder, Delusional disorder, Personality disorder unspecified, Emotionally unstable personality disorder, Paranoid schizophrenia, Bipolar affective disorder unspecified, Mental and behavioural disorders due to multiple drug use and use of other psychoactive substances
Non ST-elevation myocardial infarction	Acute myocardial infarction unspecified, Acute subendocardial myocardial infarction, Subsequent myocardial infarction
Obesity	Other obesity, Obesity unspecified, Extreme obesity with alveolar hypoventilation,
Previous myocardial infarction	Old myocardial infarction
Renal dialysis	Dependence on renal dialysis, Extracorporeal dialysis, Kidney dialysis, Mechanical complications of dialysis catheter
Renal transplant	Kidney transplant failure and rejection, Kidney transplant status
Smoking	
ST-elevation myocardial infarction	Acute transmural myocardial infarction of anterior wall, Acute transmural myocardial infarction of inferior wall, Acute transmural myocardial infarction of other sites
	Sudden cardiac death so described; Cardiac arrest unspecified, Cardiac arrest with successful resuscitation
	Transient cerebral ischaemic attack unspecified, Amaurosis fugax
Unstable angina	Unstable angina
Venous thrombo-embolism	Pulmonary embolism without mention of acute cor pulmonale, Embolism and thrombosis of lower extremities

Supplementary table 3. ICD-v10 items included in the Multimorbidity Frailty index (v10) and Hospital Frailty Risk Score.

ICD-v10 code	Description	Weighting applied	
		mFI-v10[1]	HFRS[2]
A04	Other bacterial intestinal infections	0	1.1
A09	Diarrhoea & gastroenteritis of presumed infectious origin	0	1.1
A41	Other septicaemia	0	1.6
B95	Streptococcus & staphylococcus as the cause of diseases classified to other chapters	0	1.7
B96	Other bacterial agents as the cause of diseases classified to other chapters	0	2.9

D64	Other anaemias	0	0.4
E05	Thyrotoxicosis	0	0.9
E16	Other disorders of pancreatic internal secretion	0	1.4
E53	Deficiency of other B group vitamins	0	1.9
E55	Vitamin D deficiency	0	1.0
E83	Disorders of mineral metabolism	0	0.4
E86	Volume depletion	0	2.3
E87	Disorders of electrolyte & fluid balance	1	2.3
F00	Dementia in Alzheimer's disease	0	7.1
F01	Vascular dementia	0	2.0
F03	Dementia	1	2.1
F05	Delirium, not induced by alcohol & other psychoactive substances	0	3.2
F10	mental & behavioural disorders due to use of alcohol	0	0.7
F32	Depressive episode	0	0.5
G20	Parkinson's disease	0	1.8
G30	Alzheimer's disease	0	4.0
G31	Other degenerative disorders of nervous system, not elsewhere classified	0	1.2
G40	Epilepsy	0	1.5
G45	Transient cerebral ischaemic attacks & related syndromes	0	1.2
G47	Sleep disorder & apnoea	1	0.0
G81	Hemplegia	0	4.4
H02	Disorders of eyelids	1	0.0
H35	Retinopathy & other eye disorders	1	0.0
H40	Glaucoma & other ocular hypertension	1	0.0
H54	Blindness & low vision	0	1.9
H81	Vertigo or other disorder of vestibular function	1	0.0
H91	Other hearing loss	0	0.9
I10	Hypertension	1	0.0
I11	Hypertensive heart disease with/without heart failure	1	0.0
I20	Angina	1	0.0
I25	Atherosclerotic heart disease & chronic ischaemic heart disease	1	0.0
I48	Atrial fibrillation & atrial flutter	1	0.0
I49	Cardiac arrhythmia	1	0.0
I50	Heart failure	1	0.0
I63	Cerebral infarction	1	0.8
I67	Cerebral vascular disease	1	2.6
I69	Late effect of cerebrovascular diseases	1	3.7
I95	Hypotension	0	1.6
J18	Pneumonia	1	1.1
J22	Unspecified acute lower respiratory infection	0	0.7
J44	Chronic obstructive pulmonary disease	1	0.0
J45	Asthma	1	0.0
J69	Pneumonitis due to solids & liquids	0	1.0
J96	Respiratory failure, not elsewhere classified	0	1.5

K25	Gastric ulcer	1	0.0
K26	Duodenal ulcer	0	1.6
K27	Peptic ulcer	1	0.0
K30	Functional dyspepsia	1	0.0
K52	Other noninfective gastroenteritis & colitis	0	0.3
K59	Other functional intestinal disorders	1	1.8
K92	Other diseases of digestive system	0	0.8
L03	Cellulitis	1	2.0
L08	Other local infections of skin & subcutaneous tissue	0	0.4
L30	Dermatitis	1	0.0
L89	Decubitus ulcer	0	1.7
L97	Ulcer of lower limb, not elsewhere classified	0	1.6
M10	Gout	1	0.0
M15	Polyosteoarthritis	1	0.4
M19	Osteoarthritis	1	1.5
M25	Other joint disorders, not elsewhere classified	2.3	0.0
M41	Scoliosis	0	0.9
M48	Spinal stenosis & spondyloarthropathy	1	0.5
M80	Osteoporosis with pathological fracture	0	0.8
M81	Osteoporosis	1	1.4
N17	Acute renal failure	0	1.8
N18	Chronic kidney disease	1	1.4
N19	Unspecified renal failure	0	1.6
N20	Calculus of kidney & ureter	0	0.7
N28	Other disorders of kidney & ureter, not elsewhere classified	0	1.3
N39	Other disorders of urinary system	1	3.2
N40	Enlarged & nodular prostate	1	0.0
R00	Abnormalities of heart beat	0	0.7
R02	Gangrene, not elsewhere classified	0	1.0
R05	Cough	1	0.0
R10	Abdominal pain	1	0.0
R11	Nausea & vomiting	0	0.3
R13	Dysphagia	0	0.8
R26	Abnormalities of gait & mobility	0	2.6
R29	Other symptoms & signs involving the nervous & musculoskeletal systems	0	3.6
R31	Unspecified haematuria	0	3.0
R32	Unspecified urinary incontinence	0	1.2
R33	Retention of urine	0	1.3
R40	Somnolence, stupor & coma	0	2.5
R41	Other symptoms & signs involving cognitive functions & awareness	0	2.7
R42	Dizziness & giddiness	1	0.0
R44	Other symptoms & signs involving general sensations & perceptions	0	1.6
R45	Symptoms & signs involving emotional state	0	1.2
R47	Speech disturbances, not elsewhere classified	0	1.0

R50	Fever of unknown origin	0	0.1
R54	Senility	0	2.2
R55	Syncope & collapse	0	1.8
R56	Convulsions, not elsewhere classified	0	2.6
R63	Symptoms & signs concerning food & fluid intake	0	0.9
R69	Unknown & unspecified causes of morbidity	0	1.3
R79	Other abormal findings of blood chemistry	0	0.6
R94	Abnormal results of function studies	0	1.4
S00	Superficial injury of head	0	3.2
S01	Open wound of head	0	1.1
S06	Intracranial injury	0	2.4
S09	Other & unspecified injuries of head	0	1.2
S22	Fracture of rib(s), sternum & thoracic spine	0	1.8
S32	Fracture of lumbar spine & pelvis	0	1.4
S42	Fracture of shoulder & upper arm	0	2.3
S51	Open wound of forearm	0	0.5
S72	Fracture of femur	0	1.4
S80	Superficial injury of lower leg	0	2.0
T83	Complications of genitourinary prosthetic devices, implant s& grafts	0	2.4
U80	Agent resistant to penicillin & related antibiotics	0	0.8
W01	Fall on same level from slipping, tripping & stumbling	0	0.9
W10	Fall on & from stairs & steps	0	0.9
W18	Other fall on same level	0	2.1
W19	Unspecified fall	0	3.2
X59	Exposure to unspecified factor	0	1.5
Y84	Other medical procedures as the cause of abnormal reaction of the patient	0	0.7
Y95	Nosocomial infection	0	1.2
Z22	Carrier of infectious disease	0	1.7
Z50	Care including use of rehabilitation procedures	0	2.1
Z60	Problems related to social environment	0	1.8
Z73	Problems related to life-management difficulty	0	0.6
Z75	Problems related to medical facilities & other healthcare	0	2.0
Z87	Personal history of other diseases & conditions	0	1.5
Z91	Personal history of risk-factors, not elsewhere classified	0	0.5
Z93	Artificial opening status	0	1.0
Z96	Presence of functional implant	1	0.0
Z99	Dependence on enabling machines & devices	0	0.8

[1] Multimorbidity frailty index calculated as the total sum of all deficits/ the number of deficits considered in people aged ≥65 years. mFI score of 0–0.0525 designated as fit, 0.0525–0.105 as mild frailty, 0.105–0.1575 as moderate frailty and >0.1575 as severe frailty.

[2]Hospital frailty risk score calculated as the total of the deficits weighted as shown, in people aged ≥75 years. Low risk - score of less than 5, intermediate risk (5–15), high risk (>15).

Supplementary table 4: Covariables identified "a priori" as potential effect modifiers of the association between low eGFR and reduced use of invasive management after ACS due to their potential causative association with eGFR, coronary intervention and death.

**Covariables predicted to move effect estimates further from the null**	**Covariables predicted to move effect estimates closer towards the null**
Older age<br>Female sex<br>Non-White ethnicity	Diabetes mellitus<br>Previous history of CVD

Supplementary table 5: Covariables included in the propensity score.

**Items included in the propensity score**
Age category<br>Obesity<br>Chronic obstructive pulmonary disease<br>Sex<br>Smoking status<br>Cardiovascular disease<br>Diabetes mellitus<br>Alcohol use<br>Hospital code<br>Family history of ischaemic heart disease<br>Psychiatric disorder<br>Liver disease<br>Mental health disorder<br>Malignancy<br>Ethnic category

Supplementary table 6: Covariates included in the multiple imputation model.

**Items included in the multiple imputation model**
eGFR category<br>Obesity<br>Gender<br>Smoking status<br>Age category

Previous cardiovascular disease<br>Chronic obstructive pulmonary disease<br>Diabetes mellitus<br>Ethnic category<br>Hospital code<br>Death by 30 days<br>Comorbidity count<br>Angiography during admission<br>Revascularisation during admission

Supplementary table 7: Detailed methodology for sensitivity analyses designed to investigate potential sources of bias in the association between eGFR category and coronary angiography following ACS:

Objective	Method
**To examine the impact of early death**	We used different methods to examine the impact of early death on effect estimates. We reclassified patients who died within the first 72 or 24 hours (for NSTE-ACS and STEMI respectively) as if they had all received coronary angiography (as an extreme case). We also used multivariable Cox regression analysis, rather than logistic regression, for coronary angiography, considering time to event and censoring.
**Adjusting for propensity score**	We repeated our logistic regression models adjusting for propensity scores (PS). Covariates were selected based on a confounding relationship between reduced eGFR (eGFR<60mls/min/1.73^2) and outcome (coronary intervention), or an association with the outcome only **(Supplementary table 4)**. PS were derived using logistic regression, for patients who did not die within 72 or 24 hours of initial troponin (for NSTE-ACS and STEMI respectively). The dependent variable was eGFR <60 versus ≥60mls/min/1.73^2. Patients with extreme PS were excluded from further

	analyses. We estimated the probabilities for coronary angiography using logistic regression, with adjustment for the PS.<br><br>We compared the results of our complete case analysis with those following multiple imputation of missing ethnicity data using chained equations. Covariates in the imputation model were selected if included in the substantive analysis, associated with the missing value or with the mechanism of missingness **(Supplementary table 5)**. Twenty datasets were imputed, and results combined using Rubin's rules.
**To assess robustness of our choice of frailty score**	We estimated frailty using the Hospital Frailty Risk Score(17) and comorbidity count(18) and assessed the impact of adjusting for each on the association between eGFR category and receipt of coronary angiography, stratified by ACS type **(Supplementary table 3)**.

Supplementary table 8: Table of characteristics for patients for whom a kidney test result was available (included in main analyses) and those excluded from analyses due to lack of available kidney test result.

	**Kidney test result available**<br><br>**N=10,216**	**No kidney test result available**<br>**N=56**
Age (years)	70 (59-80)	66 (59-81)
Female gender	3,081 (30.2%)	13 (23.2%)
Ethnic category		
White	6,274 (61.4%)	33 (61.2%)
Black	373 (3.7%)	5 (7.5%%)
Asian	1,059 (10.4%)	<5 (<5%)
Mixed	656 (6.4%)	<5 (<5%)
Missing	1,853 (18.2%)	15 (22.4%)
Smoking history		
Never smoked	5,794 (56.7%)	46 (82.1%)
Ex smoker	2,052 (20.1%)	5 (8.9%)
Current smoker	2,370 (23.2%)	5 (8.9%)
Diabetes mellitus	2,432 (23.8%)	10 (17.9%)
Any cardiovascular disease	8,394 (82.2%)	36 (64.3%)
Hypercholesterolaemia	3,538 (34.6%)	16 (28.6%)
Family history of IHD	1,840 (18.0%)	<5 (<5%)
Arrhythmia	1,203 (11.8%)	<5 (<5%)
Aortic stenosis	244 (2.4%)	<5 (<5%)
Congestive heart failure	1,456 (14.3%)	<5 (<5%)
Venous thrombo-embolism	33 (0.3%)	<5 (<5%)
Chronic obstructive pulmonary disease	580 (5.7%)	<5 (<5%)
Cerebrovascular event	96 (0.9%)	<5 (<5%)
Mental health disorder	2,358 (23.1%)	<5 (<5%)
Liver disease	70 (0.7%)	<5 (<5%)
Malignancy	558 (5.5%)	<5 (<5%)
Obesity	1,061 (10.4%)	<5 (<5%)

Supplementary table 9: Multivariable-adjusted odds of inpatient angiography by eGFR category and ACS type using different methods of adjustment for the competing risk of death.

ACS type	eGFR category	NSTE-ACS			STEMI		
		OR/HR	95% CI	p-value	OR/HR	95% CI	p-value
**No adjustment[1]**	>90mls/min/1.73m^2	1.00			1.00		
	60-89	1.05	0.88 - 1.26	0.60	1.21	0.85 - 1.72	0.30
	45-59	0.95	0.75 - 1.22	0.70	0.74	0.46 - 1.18	0.20
	30-44	0.74	0.56 - 0.97	0.03	0.34	0.20 - 0.57	<0.01
	<30	0.57	0.43 - 0.75	<0.01	0.25	0.15 - 0.43	<0.01
	Linear trend			<0.01			<0.01
**Early deaths dropped[1]**	>90mls/min/1.73m^2	1.00			1.00		
	60-89	1.05	0.88 - 1.27	0.58	1.20	0.84 - 1.71	0.31
	45-59	0.98	0.77 - 1.26	0.87	0.77	0.47 - 1.24	0.28
	30-44	0.76	0.57 - 1.01	0.06	0.33	0.20 - 0.56	<0.01
	<30	0.58	0.44 - 0.77	<0.01	0.28	0.16 - 0.48	<0.01
	Linear trend			<0.01			<0.01
**Early deaths treated as cases[1]**	>90mls/min/1.73m^2	1.00			1.00		
	60-89	1.02	0.85 - 1.21	0.86	1.07	0.77 - 1.49	0.69
	45-59	0.98	0.78 - 1.24	0.88	0.74	0.47 - 1.17	0.19
	30-44	0.89	0.68 - 1.17	0.41	0.36	0.22 - 0.59	<0.01
	<30	0.80	0.61 - 1.04	0.09	0.36	0.21 - 0.60	<0.01
	Linear trend			0.06			<0.01
**Cox regression[2]**	>90mls/min/1.73m^2	1.00			1.00		
	60-89	1.01	0.92 - 1.11	0.78	1.02	0.93 - 1.13	0.63
	45-59	0.89	0.78 - 1.02	0.09	0.91	0.77 - 1.07	0.26
	30-44	0.67	0.57 - 0.80	<0.01	0.72	0.58 - 0.89	<0.01
	<30	0.52	0.43 - 0.62	<0.01	0.58	0.46 - 0.74	<0.01
	Linear trend			<0.01			<0.01

[1] Expressed as odds ratio

[2] Expressed as hazard ratio

Supplementary table 10. Comparison of effect estimates for the odds of inpatient angiography for people with eGFR<60mls/min/1.73m^2 versus those with an eGFR≥60 between multivariable and propensity score adjusted models, by ACS type.

ACS type	eGFR category	Multivariable-adjusted			Propensity score-adjusted		
		OR	95% CI	p-value	OR	95% CI	p-value
**NSTE-ACS**	Normal eGFR	1			1		
	eGFR<60	0.76	0.65 - 0.87	<0.01	0.77	0.67-0.89	<0.01
**STEMI**	Normal eGFR	1			1		
	eGFR<60	0.41	0.30-0.55	<0.01	0.52	0.39-0.69	<0.01

Supplementary table 11: Comparison of the multivariable-adjusted odds of inpatient angiography by eGFR category and ACS type using a complete case analysis versus multiple imputation of missing ethnicity data.

ACS type	eGFR category	Complete case analysis			Multiple imputation for ethnic category		
		**OR/HR**	**95% CI**	**p-value**	**OR/HR**	**95% CI**	**p-value**
**NSTE-ACS**	>90mls/min/1.73m^2	1.00			1.00		
	60-89	1.05	0.88 - 1.27	0.58	1.07	0.91 - 1.27	0.40
	45-59	0.98	0.77 - 1.26	0.87	0.94	0.75 - 1.17	0.57
	30-44	0.76	0.57 - 1.01	0.06	0.79	0.61 - 1.03	0.08
	<30	0.58	0.44 - 0.77	<0.01	0.56	0.43 - 0.74	<0.01
	Linear trend			<0.01			<0.01
**STEMI**	>90mls/min/1.73m^2	1.00			1.00		
	60-89	1.20	0.84 - 1.71	0.31	1.03	0.77 - 1.38	0.85
	45-59	0.77	0.47 - 1.24	0.28	0.75	0.49 - 1.14	0.18
	30-44	0.33	0.20 - 0.56	<0.01	0.42	0.26 - 0.66	<0.01
	<30	0.28	0.16 - 0.48	<0.01	0.35	0.21 - 0.56	<0.01
	Linear trend			<0.01			<0.01

Supplementary table 12: Multivariable-adjusted odds of inpatient angiography by eGFR category and ACS type, with adjustment for Multimorbidity frailty index, Hospital frailty risk score or comorbidity count.

ACS type	eGFR category	NSTE-ACS			STEMI		
		OR	95% CI	p-value	OR	95% CI	p-value
No adjustment for frailty	>90mls/min/1.73m^2	1.00			1.00		
	60-89	1.05	0.88 - 1.27	0.58	1.20	0.84 - 1.71	0.31
	45-59	0.98	0.77 - 1.26	0.87	0.77	0.47 - 1.24	0.28
	30-44	0.76	0.57 - 1.01	0.06	0.33	0.20 - 0.56	<0.01
	<30	0.58	0.44 - 0.77	<0.01	0.28	0.16 - 0.48	<0.01
	Linear trend			<0.01			<0.01
Adjusted for mFI[1]	>90mls/min/1.73m^2	1.00			1.00		
	60-89	1.05	0.88 - 1.27	0.57	1.20	0.84 - 1.71	0.31
	45-59	0.98	0.76 - 1.25	0.85	0.77	0.47 - 1.24	0.28
	30-44	0.75	0.57 - 1.00	0.05	0.33	0.20 - 0.56	<0.01
	<30	0.57	0.43 - 0.76	<0.01	0.28	0.16 - 0.49	<0.01
	Linear trend			<0.01			<0.01
Adjusted for HFRS[2]	>90mls/min/1.73m^2	1.00			1.00		
	60-89	1.02	0.85 - 1.23	0.83	1.18	0.83 - 1.68	0.36
	45-59	0.97	0.76 - 1.24	0.81	0.78	0.48 - 1.27	0.32
	30-44	0.78	0.59 - 1.04	0.09	0.36	0.21 - 0.61	<0.01
	<30	0.65	0.49 - 0.87	<0.01	0.36	0.20 - 0.64	<0.01
	Linear trend			<0.01			<0.01
Adjusted for comorbidity count	>90mls/min/1.73m^2	1.00			1.00		
	60-89	1.06	0.88 - 1.27	0.55	1.23	0.86 - 1.76	0.25
	45-59	0.99	0.77 - 1.27	0.93	0.82	0.50 - 1.33	0.42
	30-44	0.76	0.58 - 1.02	0.06	0.37	0.22 - 0.63	<0.01
	<30	0.59	0.44 - 0.78	<0.01	0.32	0.19 - 0.56	<0.01
	Linear trend			<0.01			<0.01

[1] Multimorbidity Frailty Index; [2]Hospital Frailty Risk Score

Supplementary table 13: Multivariable-adjusted odds of inpatient angiography by ACS type, with and without adjustment for clustering at the hospital level.

ACS type	eGFR category	Unadjusted			Adjusted for clustering by hospital		
		OR	95% CI	p-value	OR	95% CI	p-value
NSTE-ACS	>90mls/min/1.73m^2	1.00			1.00		
	60-89	1.05	0.88 - 1.27	0.58	1.05	0.89 - 1.24	0.53
	45-59	0.98	0.77 - 1.26	0.87	0.98	0.85 - 1.13	0.78
	30-44	0.76	0.57 - 1.01	0.06	0.76	0.55 - 1.06	0.11
	<30	0.58	0.44 - 0.77	<0.01	0.58	0.46 - 0.73	<0.01
	Linear trend			<0.01			<0.01
STEMI	>90mls/min/1.73m^2	1.00			1.00		
	60-89	1.20	0.84 - 1.71	0.31	1.20	1.05 - 1.37	0.01
	45-59	0.77	0.47 - 1.24	0.28	0.77	0.54 - 1.08	0.13
	30-44	0.33	0.20 - 0.56	<0.01	0.33	0.28 - 0.39	<0.01
	<30	0.28	0.16 - 0.48	<0.01	0.28	0.23 - 0.34	<0.01
	Linear trend			<0.01			<0.01

Supplementary table 14: Multivariable-adjusted odds ratios for inpatient angiography adjusted for composite CVD variable versus multiple distinct CVD-related variables

ACS type	eGFR category	Composite CVD variable[1]			Individual CVD variables[2]		
		**OR**	**95% CI**	**p-value**	**OR**	**95% CI**	**p-value**
**NSTE-ACS**	>90mls/min/1.73m^2	1.00			1.00		
	60-89	1.05	0.88 - 1.27	0.58	1.02	0.84 - 1.23	0.86
	45-59	0.98	0.77 - 1.26	0.87	0.98	0.76 - 1.27	0.90
	30-44	0.76	0.57 - 1.01	0.06	0.83	0.61 - 1.11	0.20
	<30	0.58	0.44 - 0.77	<0.01	0.60	0.45 - 0.80	<0.01
	Linear trend			<0.01			<0.01
**STEMI**	>90mls/min/1.73m^2	1.00			1.00		
	60-89	1.20	0.84 - 1.71	0.31	1.19	0.83 - 1.71	0.35
	45-59	0.77	0.47 - 1.24	0.28	0.80	0.48 - 1.33	0.39
	30-44	0.33	0.20 - 0.56	<0.01	0.30	0.17 - 0.53	<0.01
	<30	0.28	0.16 - 0.48	<0.01	0.29	0.16 - 0.52	<0.01
	Linear trend			<0.01			<0.01

[1] Composite CVD variable includes ischaemic heart disease, peripheral vascular disease, aortic aneurysm, ischaemic cerebrovascular event, haemorrhagic cerebrovascular event, transient ischaemic attack, prior myocardial infarction, congestive heart failure, stable angina.

[2]Above items included as individual covariates in model

Supplementary table 15: Multivariable-adjusted odds ratios for inpatient angiography without and with inclusion of people with a first troponin value recorded within the 24 hours following a coronary intervention.

ACS type	eGFR category	Post procedure troponin excluded			Post procedure troponin included		
		OR	95% CI	p-value	OR	95% CI	p-value
NSTE-ACS	>90mls/min/1.73m^2	1			1		
	60-89	1.07	0.91 - 1.27	0.42	1.06	0.88 - 1.27	0.54
	45-59	0.94	0.75 - 1.18	0.59	1	0.78 - 1.28	1
	30-44	0.79	0.61 - 1.02	0.07	0.8	0.60 - 1.06	0.12
	<30	0.56	0.43 - 0.73	<0.01	0.63	0.47 - 0.83	<0.01
	Linear trend						
STEMI	>90mls/min/1.73m^2	1			1		
	60-89	1.03	0.77 - 1.39	0.82	1.19	0.83 - 1.69	0.34
	45-59	0.77	0.50 - 1.17	0.22	0.77	0.48 - 1.23	0.27
	30-44	0.42	0.27 - 0.66	<0.01	0.33	0.20 - 0.56	<0.01
	<30	0.35	0.21 - 0.56	<0.01	0.29	0.17 - 0.50	<0.01
	Linear trend						

Supplementary table 16: Multivariable-adjusted odds of angiography by eGFR category and ACS type, with and without inclusion of patients with a code for revascularization but not for angiography

ACS type	eGFR category	Assuming revascularized patients received angiography			Exclusion of revascularized patients without angiography		
		OR	95% CI	p-value	OR	95% CI	p-value
**NSTE-ACS**	>90mls/min/1.73m^2	1.00			1.00		
	60-89	1.05	0.88 - 1.27	0.58	1.05	0.87 - 1.27	0.60
	45-59	0.98	0.77 - 1.26	0.87	0.98	0.76 - 1.26	0.86
	30-44	0.76	0.57 - 1.01	0.06	0.76	0.57 - 1.02	0.07
	<30	0.58	0.44 - 0.77	<0.01	0.59	0.44 - 0.79	<0.01
	Linear trend			<0.01			<0.01
**STEMI**	>90mls/min/1.73m^2	1.00			1.00		
	60-89	1.20	0.84 - 1.71	0.31	1.19	0.84 - 1.70	0.33
	45-59	0.77	0.47 - 1.24	0.28	0.77	0.48 - 1.24	0.28
	30-44	0.33	0.20 - 0.56	<0.01	0.32	0.19 - 0.55	<0.01
	<30	0.28	0.16 - 0.48	<0.01	0.27	0.16 - 0.47	<0.01
	Linear trend			<0.01			<0.01

Supplementary table 17: Multivariable-adjusted odds ratios for inpatient angiography and revascularisation by eGFR category for people with NSTE-ACS with and without inclusion of those with unstable angina.

Outcome	eGFR category	Inclusion of unstable angina			Exclusion of unstable angina		
		OR	95% CI	p-value	OR	95% CI	p-value
Angio	>90mls/min/1.73m^2	1.00			1.00		
	60-89	1.05	0.88 - 1.27	0.58	1.07	0.84 - 1.36	0.60
	45-59	0.98	0.77 - 1.26	0.87	0.78	0.58 - 1.06	0.12
	30-44	0.76	0.57 - 1.01	0.06	0.64	0.46 - 0.91	0.01
	<30	0.58	0.44 - 0.77	<0.01	0.40	0.29 - 0.56	<0.01
	Linear trend			<0.01			<0.01
Revascularisation	>90mls/min/1.73m^2	1.00			1.00		
	60-89	0.98	0.83 - 1.17	0.83	0.97	0.78 - 1.21	0.81
	45-59	0.82	0.65 - 1.04	0.10	0.69	0.52 - 0.92	0.01
	30-44	0.62	0.47 - 0.82	<0.01	0.58	0.42 - 0.81	<0.01
	<30	0.53	0.40 - 0.70	<0.01	0.40	0.29 - 0.55	<0.01
	Linear trend			<0.01			<0.01

Supplementary table 18: Multivariable-adjusted odds ratios for revascularisation by eGFR category and ACS type, stratified by age group.

ACS type	eGFR category	< 65 years				65 - 75 years				>75 years			
		**N**	**OR**	**95% CI**	**p-value**	**N**	**OR**	**95% CI**	**p-value**	**N**	**OR**	**95% CI**	**p-value**
**NSTE-ACS**	≥90mls/min/1.73m^2	1,074	1			333	1			63	1		
	60-89	442	1.08	0.85 - 1.37	0.55	737	0.79	0.59 - 1.05	0.11	1,077	1.08	0.65 - 1.78	0.77
	45-59	59	0.83	0.49 - 1.44	0.51	136	0.70	0.45 - 1.09	0.12	496	0.74	0.44 - 1.24	0.25
	30-44	31	0.35	0.16 - 0.77	0.01	73	0.41	0.23 - 0.72	<0.01	334	0.59	0.35 - 1.02	0.06
	<30	50	0.63	0.35 - 1.13	0.12	109	0.50	0.30 - 0.81	0.01	237	0.46	0.26 - 0.81	0.01
		**N**	**OR**	**95% CI**	**p-value**	**N**	**OR**	**95% CI**	**p-value**	**N**	**OR**	**95% CI**	**p-value**
**STEMI**	≥90mls/min/1.73m^2	976	1			249	1			25	1		
	60-89	375	1.04	0.73 - 1.47	0.85	420	1.24	0.78 - 1.98	0.36	436	2.74	1.12 - 6.69	0.03
	45-59	48	1.42	0.58 - 3.49	0.44	66	0.89	0.42 - 1.86	0.75	135	2.00	0.77 - 5.14	0.15
	30-44	24	0.26	0.11 - 0.63	<0.01	24	0.24	0.09 - 0.62	<0.01	100	1.33	0.50 - 3.49	0.57
	<30	20	0.77	0.24 - 2.44	0.65	22	0.22	0.08 - 0.58	<0.01	83	1.33	0.50 - 3.58	0.57

Supplementary table 19: Multivariable-adjusted odds ratios for revascularisation after STEMI comparing eGFR>60mls/min/1.73m2 to eGFR categories <60, stratified by age group.

ACS type	eGFR category	< 65 years				65 - 75 years				>75 years			
		**N**	**OR**	**95% CI**	**p-value**	**N**	**OR**	**95% CI**	**p-value**	**N**	**OR**	**95% CI**	**p-value**
**STEMI**	**≥60mls/min/1.73m^2**	1,351	1			669	1			461	1		
	45-59	48	1.4	0.58 - 3.42	0.46	66	0.77	0.39-1.52	0.45	135	0.78	0.49 - 1.23	0.28
	30-44	24	0.26	0.11 - 0.62	<0.01	24	0.21	0.09 - 0.51	<0.01	100	0.52	0.31 - 0.86	0.01
	<30	20	0.76	0.24 - 2.40	0.64	22	0.19	0.07 - 0.48	<0.01	83	0.52	0.30 - 0.89	0.02

Supplementary table 19: STROBE Reporting Checklist

	Item No.	Recommendation	Page No.
	1	(a) Indicate the study's design with a commonly used term in the title or the abstract<br>(b) Provide in the abstract an informative and balanced summary of what was done and what was found	2
**Introduction**			
Background/rationale	2	Explain the scientific background and rationale for the investigation being reported	5
Objectives	3	State specific objectives, including any prespecified hypotheses	6
**Methods**			
Study design	4	Present key elements of study design early in the paper	6
Setting	5	Describe the setting, locations, and relevant dates, including periods of recruitment, exposure, follow-up, and data collection	6
Participants	6	(*a*) *Cohort study*—Give the eligibility criteria, and the sources and methods of selection of participants. Describe methods of follow-up<br>*Case-control study*—Give the eligibility criteria, and the sources and methods of case ascertainment and control selection. Give the rationale for the choice of cases and controls<br>*Cross-sectional study*—Give the eligibility criteria, and the sources and methods of selection of participants	6-7
		(*b*) *Cohort study*—For matched studies, give matching criteria and number of exposed and unexposed<br>*Case-control study*—For matched studies, give matching criteria and the number of controls per case	
Variables	7	Clearly define all outcomes, exposures, predictors, potential confounders, and effect modifiers. Give diagnostic criteria, if applicable	6-7
Data sources/ measurement	8*	For each variable of interest, give sources of data and details of methods of assessment (measurement). Describe comparability of assessment methods if there is more than one group	6-7
Bias	9	Describe any efforts to address potential sources of bias	9
Study size	10	Explain how the study size was arrived at	6
Quantitative variables	11	Explain how quantitative variables were handled in the analyses. If applicable, describe which groupings were chosen and why	7
Statistical methods	12	(*a*) Describe all statistical methods, including those used to control for confounding	8-9

		(*b*) Describe any methods used to examine subgroups and interactions	9
		(*c*) Explain how missing data were addressed	9
		(*d*) *Cohort study*—If applicable, explain how loss to follow-up was addressed<br>*Case-control study*—If applicable, explain how matching of cases and controls was addressed<br>*Cross-sectional study*—If applicable, describe analytical methods taking account of sampling strategy	N/a
		(*e*) Describe any sensitivity analyses	9
Participants	13*	(a) Report numbers of individuals at each stage of study—eg numbers potentially eligible, examined for eligibility, confirmed eligible, included in the study, completing follow-up, and analysed	9 (Also S. Table 1)
		(b) Give reasons for non-participation at each stage	S. Table 1
		(c) Consider use of a flow diagram	S. Table 1
Descriptive data	14*	(a) Give characteristics of study participants (eg demographic, clinical, social) and information on exposures and potential confounders	Table 1
		(b) Indicate number of participants with missing data for each variable of interest	Table 1
		(c) *Cohort study*—Summarise follow-up time (eg, average and total amount)	10
Outcome data	15*	*Cohort study*—Report numbers of outcome events or summary measures over time	Tables 2-5
		Case-control study—Report numbers in each exposure category, or summary measures of exposure	
		Cross-sectional study—Report numbers of outcome events or summary measures	
Main results	16	(*a*) Give unadjusted estimates and, if applicable, confounder-adjusted estimates and their precision (eg, 95% confidence interval). Make clear which confounders were adjusted for and why they were included	Tables 2-5
		(*b*) Report category boundaries when continuous variables were categorized	6
		(*c*) If relevant, consider translating estimates of relative risk into absolute risk for a meaningful time period	Tables 2,4
Other analyses	17	Report other analyses done—eg analyses of subgroups and interactions, and sensitivity analyses	13
Key results	18	Summarise key results with reference to study objectives	13-14

Limitations	19	Discuss limitations of the study, taking into account sources of potential bias or imprecision. Discuss both direction and magnitude of any potential bias	16
Interpretation	20	Give a cautious overall interpretation of results considering objectives, limitations, multiplicity of analyses, results from similar studies, and other relevant evidence	16
Generalisability	21	Discuss the generalisability (external validity) of the study results	15
**Other information**			
Funding	22	Give the source of funding and the role of the funders for the present study and, if applicable, for the original study on which the present article is based	18

Figures

Supplementary figure 1: Flow chart of inclusion/exclusion

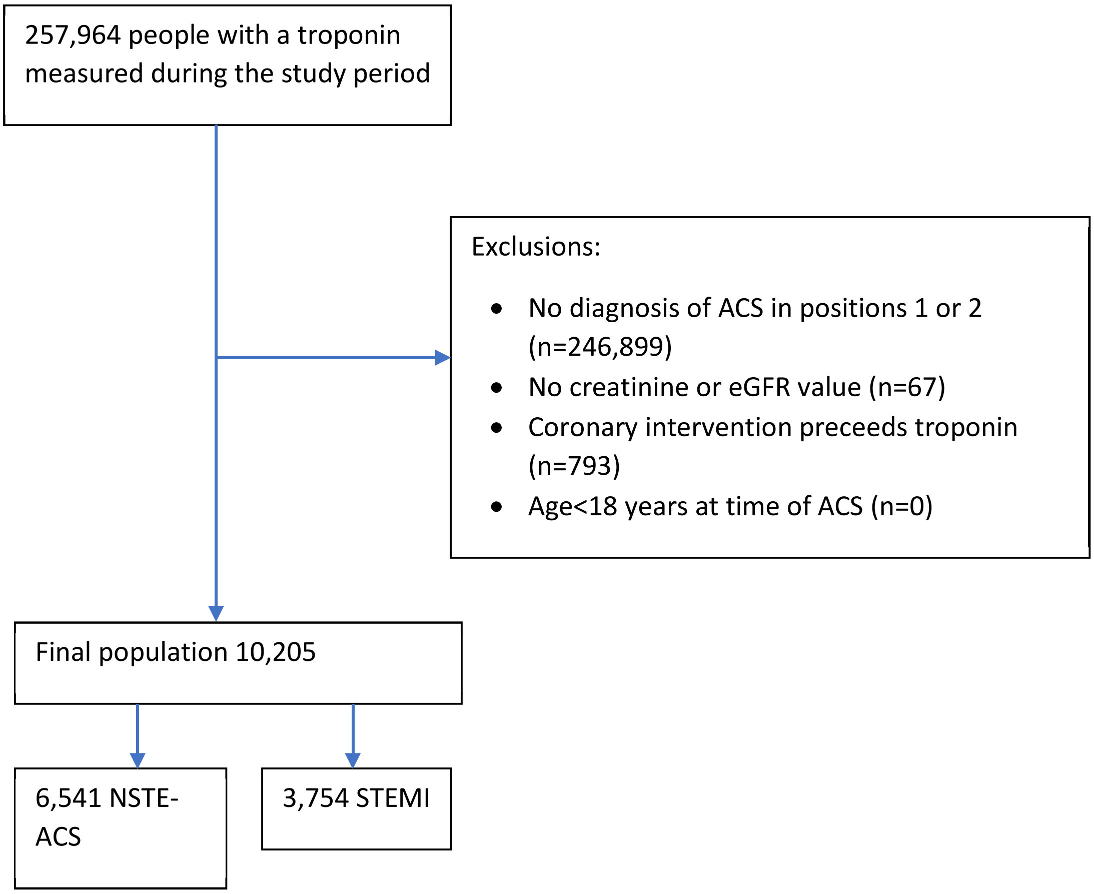

Supplement: online supplemental file 1 [file openhrt-11-2-s001.pdf]
